# Intravaginal progesterone as a fertility-sparing treatment for symptomatic adenomyosis: preliminary results from a monocentric study

**DOI:** 10.1007/s00404-026-08361-y

**Published:** 2026-04-28

**Authors:** Carlo De Cicco Nardone, Maria Cristina Sangiovanni, Gian Mario Sangiovanni, Francesco Plotti, Roberto Montera, Daniela Luvero, Roberto Angioli, Corrado Terranova

**Affiliations:** 1 Campus Bio-Medico University Hospital of Rome, Department of Obstetrics and Gynecology, Rome, Italy; 2 Sapienza University of Rome, Rome, Italy

**Keywords:** Adenomyosis, Chronic pelvic pain, Dysmenorrhea, Fertility preservation, Intravaginal progesterone

## Abstract

**Purpose:**

Adenomyosis is a chronic uterine disorder characterised by ectopic endometrial tissue within the myometrium, frequently associated with dysmenorrhea, abnormal uterine bleeding (AUB), chronic pelvic pain (CPP), and dyspareunia. No standardised guidelines are currently available for its management, and therapeutic options remain limited for women seeking fertility preservation. This study aimed to evaluate the efficacy of intravaginal progesterone in alleviating adenomyosis-related symptoms in patients seeking fertility preservation.

**Methods:**

In this prospective monocentric observational study, 85 patients aged 22–50 years with ultrasound-confirmed symptomatic adenomyosis were enrolled between April 2020 and April 2024. Inclusion criteria were age 18–55 years, BMI 18–35, and a Visual Analogue Scale (VAS) score ≥ 7 for dysmenorrhea, AUB, CPP, or dyspareunia. All patients received 200 mg/day of intravaginal progesterone for 10 days per cycle. Symptom severity was assessed using VAS scores at baseline and at 6 months. Data were analysed using non-parametric statistical tests.

**Results:**

Sixty-five patients completed the 6-month follow-up. Four patients conceived during treatment and were excluded from the final analysis. Statistically significant improvements were observed for all assessed symptoms (all p < 0.05). Median VAS scores decreased for dysmenorrhea (9 to 6, p < 0.001), AUB (8 to 6, p < 0.001), chronic pelvic pain (5.5 to 3.5, p < 0.001), and dyspareunia (2 to 0, p = 0.020). The overall treatment satisfaction was high, with a mean Likert score of 7.5 out of 10.

**Conclusion:**

Intravaginal progesterone appears to be an effective fertility-sparing treatment for symptomatic adenomyosis, providing significant relief across all primary symptoms, with high patient satisfaction. Larger controlled studies are warranted to confirm these preliminary findings and further define its role in clinical practice.

## What does this study add to the clinical work

**Table:** 

Intravaginal progesterone emerged as a simple, well-tolerated therapy that significantly improves key symptoms of adenomyosis without compromising fertility. This approach provides a practical fertility-sparing option that can be incorporated into individualised management strategies.	

## Introduction

Adenomyosis is a benign uterine condition, characterised by the abnormal growth of endometrial glands and the stroma in the myometrium of the uterus [1]. Adenomyosis, which presents with debilitating symptoms, significantly impacts various aspects of life [2]. It is estimated that one in 10 women with subfertility has a diagnosis of isolated adenomyosis [3].

Hyperestrogenism has been proven to be the crucial hormone driving the growth of endometrial glands of adenomyosis [4], overpowering the effects of progesterone [5]. Estrogenic-dependent ectopic sites of endometrium into the myometrium present a high density of nerve fibres, responsible for the development of chronic pelvic pain (CPP), dysmenorrhea and dyspareunia [6]. Moreover, in adenomyotic uteri, progesterone fails to counteract local hyperestrogenism, leading to pain symptoms and abnormal uterine bleeding [4].

Combined oral contraceptives (COCs) are considered the first-line treatment for dysmenorrhea and menorrhagia (abnormal uterine bleeding—AUB) due to their ability to induce amenorrhea through decidualization and atrophy of the endometrium [7]. Nonetheless, many patients are unsuitable for this hormonal therapy [8], and the occurrence of side effects leads patients to poor adherence to treatment [9]. Moreover, the desire for pregnancy leads some patients to avoid COCs [10].

The levonorgestrel intrauterine system (LNG-IUD), another hormonal therapy used off-label for adenomyosis symptoms [11], is still a contraceptive method [12]. The use of local progesterone via the LNG-IUD for adenomyosis-related pain is well supported in the literature, as it acts directly on adenomyotic foci and modulates altered endometrial factors [13].

The treatment of adenomyosis should be personalised [13]. Nevertheless, no guidelines are available to direct the management of adenomyosis [14]. Therefore, to date, the scarcity of high-quality studies leaves women ineligible for hormonal therapy and/or seeking fertility-preserving options with limited choices [14].

Local Vaginal progesterone is a therapy compatible with the desire for pregnancy, as it is used extensively in obstetric pathology, i.e. for the prevention of preterm birth [15, 16] or for threatened miscarriage [17] and as luteal phase support in assisted reproductive technology cycles [18].

To our knowledge, no previous studies have specifically evaluated the use of vaginal progesterone for the management of dysmenorrhea, AUB, chronic pelvic pain and dyspareunia [19].

This study aims to report the findings of local intravaginal progesterone therapy in adenomyotic patients who are not candidates for hormone therapy and who desire to retain fertility.

## Materials and methods

This study is a prospective observational study conducted at the Gynaecological Unit of the Campus Bio-Medico University Hospital in Rome. The study involved patients with symptomatic adenomyosis between April 2020 and April 2024. The study adhered to the Strengthening the Reporting of Observational Studies in Epidemiology (STROBE) guidelines [20].

Eighty-five patients aged between 22 and 50 years were enrolled in the study. Inclusion criteria for the study protocol included age 18–50 years, BMI between 18–35, regular menstrual cycles (defined as 24–38 days with ≤ 7–9 days variation, according to FIGO criteria [21, 22]), and symptoms of adenomyosis: dysmenorrhea, AUB and CPP evaluated with a score based on a decimal VAS scale of 7 or higher. Only patients with a confirmed diagnosis of adenomyosis on transvaginal ultrasound, based on the Morphological Uterus Sonographic Assessment (MUSA) [23] criteria and the modified Delphi procedure [24], were included.

Exclusion criteria included the presence of oncological conditions, pregnancy or breastfeeding, and patients undergoing surgical treatment during the medical intervention.

Written informed consent was obtained from the patient. The internal Ethics Committee of our Hospital approved this study. The study was conducted in accordance with the ethical standards outlined in the 1964 Declaration of Helsinki and its subsequent amendments, or with comparable ethical standards.

The initial evaluation consisted of a comprehensive medical history, physical examination, and transvaginal ultrasound. As part of the medical history, the regularity of the menstrual cycle was assessed based on the patient's self-reported cycle length and variability. Regular menstrual cycles were defined according to FIGO guidelines [21, 22] as cycles lasting 24–38 days with variation between cycles not exceeding 7–9 days. During the gynaecological physical examination, conducted via bimanual palpation, the size, mobility, and tenderness of the uterus were assessed. Diagnosis of adenomyosis was made by Transvaginal US (TVUS), according to the Morphological Uterus Sonographic Assessment (MUSA) [23] features of uterine Adenomyosis and to the modified Delphi procedure [24].

Quantitative variables were handled as follows: symptoms (dysmenorrhea, AUB, dyspareunia, CPP) were rated on a decimal VAS [25] scale from 0 to 10 before beginning treatment. Symptoms were grouped and analysed for severity before and after treatment at three- and six-month follow-up. During the 6-month follow-up visit, patients were asked to rate their satisfaction with improvement in quality of life using a VAS scale from 0 to 10. Additionally, careful counselling on dietary guidelines was provided at every check-up, advising patients to follow a diet rich in fibre and to reduce their consumption of red meat and saturated fats.

Methods used to examine the population were detailed. Missing data were addressed appropriately. For cohort studies, the reasons for loss to follow-up were explained. Sensitivity analyses were conducted to ensure robustness of the results.

All patients included were treated by micronized natural Progesterone, administered intravaginally in the form of vaginal tablets, at a dosage of 200 mg/day (1 vaginal capsule/day at bedtime) from the 17th to the 26th day of their cycle. Symptoms were evaluated at 6 months to assess the treatment's ongoing effectiveness. Overall satisfaction, measured on a Likert scale (0–10 points, where 10 represents the maximum satisfaction), with the therapy's impact on quality of life was also evaluated at the 6-month follow-up.

The design and reporting adhered to the STROBE (Strengthening the Reporting of Observational Studies in Epidemiology) guidelines [26]. No formal sample size or power calculation was performed. Statistical analysis was conducted using a 95% confidence level. Data distribution was first assessed using the Shapiro–Wilk test, which rejected the null hypothesis of normality for all variables. Therefore, the Wilcoxon signed-rank test, appropriate for paired non-normally distributed data, was used to compare pre- and post-treatment scores for each symptom. Within-subject changes were estimated using the Hodges–Lehmann method, and 95% confidence intervals for the median differences were calculated.

## Results

**Fig. 1:**
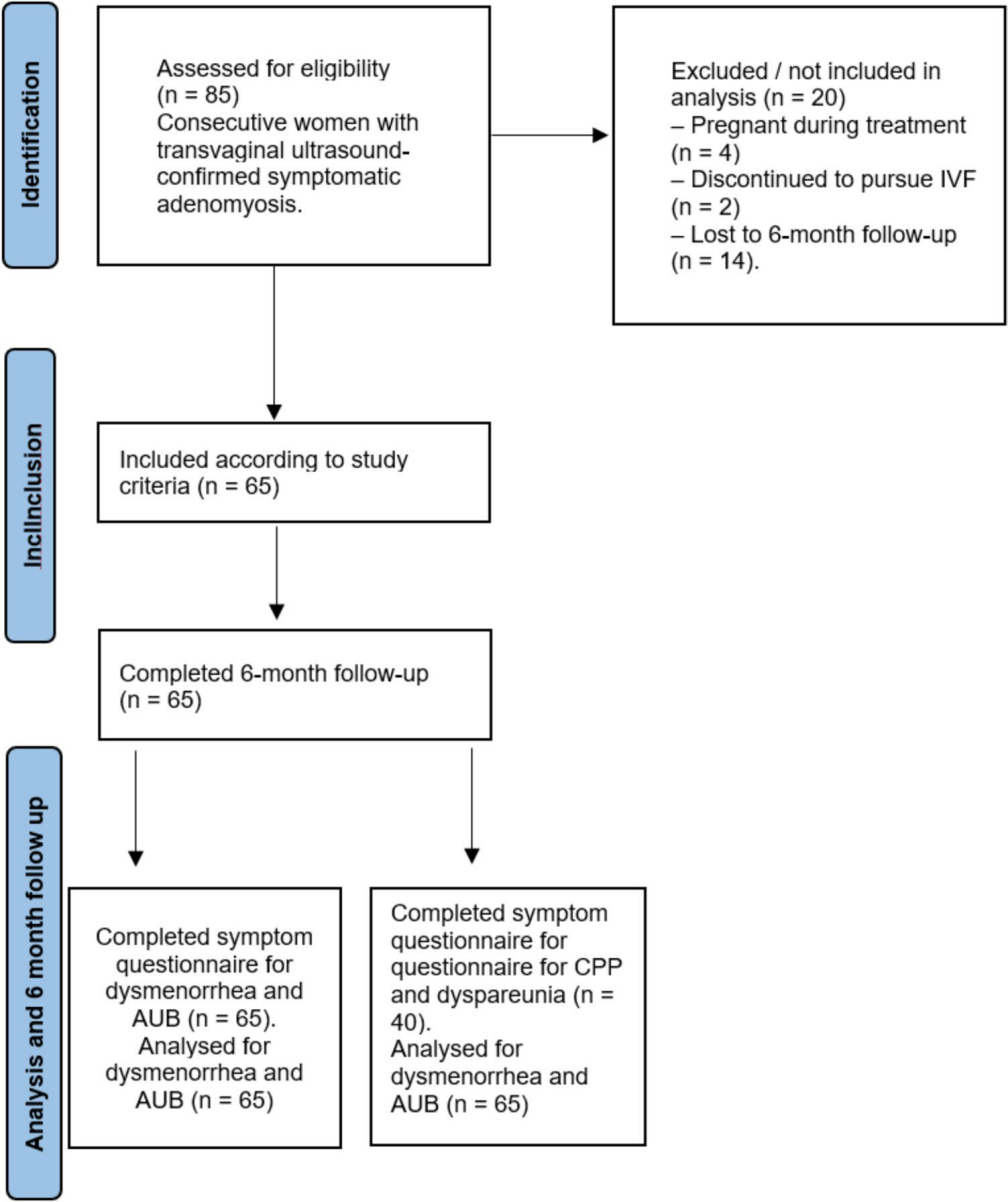
STROBE flow chart of patient screening, inclusion, analysis and follow-up. *AUB* abnormal uterine bleeding; *CPP* chronic pelvic pain; *IVF* in vitro fertilization

Of the 85 patients enrolled, 65 completed the study. Twenty patients did not complete the study. Four among these patients achieved pregnancy during treatment, two dropped out to seek pregnancy through medically assisted procreation techniques; The remaining 14 patients did not perform follow-up visits at 6 months (Fig. 1).

Clinical characteristics investigated included dysmenorrhea, AUB, CPP and dyspareunia, with transvaginal ultrasound-confirmed adenomyosis. All patients completed the VAS questionnaires for dysmenorrhea and AUB; therefore, statistical analysis for these symptoms was performed on the entire cohort of 65 patients. Conversely, only 40 patients completed the questionnaires related to dyspareunia and CPP, and consequently, the analysis for these two symptoms was limited to 40 patients. At the 6-month follow-up, all 65 patients were also asked about their satisfaction with the treatment (Fig. 1).

Baseline demographic characteristics of the study population are reported in Table 1.

**Table 1:** Demographic characteristics of the study population

	Mean	Standard deviation (SD)	Number of patients (%)
Age (years)	35.34	8.31	65
Body Mass Index (BMI)	22.62	2.28	65
Nulliparity	–	–	49 (75.4)
Parity			
1 Previous Pregnancy	–	–	9 (13.8)
≥ 2 Previous Pregnancies	–	–	7 (10.8)

VAS scores for dysmenorrhea, AUB, CPP, and dyspareunia all decreased significantly from baseline to 6 months. As shown in Table 2, median scores improved for all symptoms: dysmenorrhea decreased from 9 to 6 (p < 0.001), menorrhagia from 8 to 6 (p < 0.001), CPP from 5.5 to 3.5 (p < 0.001), and dyspareunia from 2 to 0 (p = 0.020).

**Table 2:** Pre- and post-treatment symptom scores assessed by Visual Analogue Scale (VAS). Values are expressed as medians. Statistical significance was assessed using the Wilcoxon signed-rank test for paired data. 95% Confidence Intervals (CI) for the median difference were calculated using the Hodges–Lehmann method

Symptom	N. of patients	Median VAS score before treatment	Median VAS score after 6 months	95% CI for median difference	P-value
Dysmenorrhea	65	9	6	[2.5, 3.0]	< 0.001
Menorrhagia-AUB	65	8	6	[2.0, 2.5]	< 0.001
Chronic pelvic pain- CPP	40	5.5	3.5	[2.0, 4.5]	< 0.001
Dyspareunia	40	2	0	[1.0, 4.0]	0.020

The median within-subject reductions, estimated using the Hodges–Lehmann method, were 2.5 points for dysmenorrhea, 2.0 for menorrhagia, 3.0 for chronic pelvic pain, and 2.5 for dyspareunia. The 95% confidence intervals for the median differences confirmed that the changes were consistently positive, indicating clinically meaningful improvements across all assessed symptoms.

These findings support the effectiveness of the treatment regimen in alleviating the most prevalent symptoms of adenomyosis and improving patients’ quality of life.

The overall satisfaction rating for the treatment's impact on quality of life was 7.5/10, using the Likert scale [25] at a 6-month follow-up. These results indicate a significant reduction in symptom severity across all evaluated parameters.

## Discussion

This prospective monocentric observational study suggests that a cyclic regimen of intravaginal micronized natural progesterone, administered intravaginally in the form of vaginal tablets, at a dosage of 200 mg/day (1 vaginal capsule/day at bedtime), may be associated with a clinical improvement in adenomyosis-related symptoms over a 6-month follow-up period in women seeking fertility preservation.

Statistically significant reductions were observed across all primary symptomatic clusters, including dysmenorrhea, AUB, CPP, and dyspareunia. Overall patient satisfaction with treatment was high, with a mean score of 7.5 out of 10, indicating good acceptability of the therapeutic regimen. These results align with the study’s objectives to provide effective symptom management in a patient population with limited non-contraceptive treatment options.

At present, the medical management of adenomyosis remains challenging, as no standardized clinical guidelines are available [13]. Moreover, to our knowledge, no previous studies have specifically evaluated the use of vaginal progesterone for the management of dysmenorrhea, AUB, CPP, and dyspareunia in women with symptomatic adenomyosis.

A recent systematic review and network meta-analysis by Feng et al. (2023) [27] identified Dienogest, derived from progesterone, as the most effective treatment option for reducing adenomyosis-associated CPP at 3 and 6 months. These findings support the central role of progestins in managing symptoms associated with adenomyosis and provide a benchmark for interpreting the potential effectiveness of vaginal progesterone.

From a biological perspective, progesterone signalling in adenomyotic tissue appears to be impaired, a phenomenon commonly referred to as progesterone resistance [13]. Clinical evidence suggests that high local concentrations of progesterone may overcome progesterone resistance, providing a strong biological rationale for locally delivered progesterone-based therapies [13].

Consistent with this concept, both Ota et al. [28] and Vannuccini et al. [29] demonstrated the effectiveness of the LNG-IUD system in managing adenomyosis-related pain and AUB, highlighting the key role of sustained local uterine progestin exposure in symptom modulation. However, LNG-IUD systems are intrinsically contraceptive, limiting their suitability for women seeking fertility preservation.

In this context, alternative locally administered progesterone-based approaches, such as micronized natural vaginal progesterone, warrant investigation as potential fertility-sparing options. Compared with oral administration, vaginal progesterone also ensures uterine uptake with minimal systemic effects and low plasma progesterone levels [30]. Through decidualization and atrophy of ectopic endometrial tissue, modulation of inflammatory pathways, reduction of vascularization to adenomyotic lesions and the reduction of estrogen-driven effects [31], locally administered progesterone may contribute to the improvement of adenomyosis-related pain and bleeding[14].

In line with these mechanisms, the present study demonstrated a statistically significant reduction across all assessed symptoms—dysmenorrhea, AUB, CPP, and dyspareunia—following treatment with intravaginal progesterone. These findings suggest that the enhanced local uterine exposure provided by the vaginal route may be the primary driver behind the clinically meaningful symptom relief observed in our cohort.

Notably, dyspareunia also showed a significant improvement (p = 0.020), with median scores decreasing from 2 to 0. While dyspareunia is often considered a multifactorial symptom involving pelvic floor dysfunction and neuropsychological components rather than being driven solely by uterine pathology [32], our results indicate that targeted hormonal treatment of adenomyosis can contribute to its relief. However, the slightly higher p-value compared to other symptoms (p < 0.001 for dysmenorrhea and AUB) may still reflect the complex nature of this specific pain.

Overall, these findings underscore the importance of a patient-centred and fertility-sparing therapeutic strategy, particularly in women for whom standard hormonal treatments may be contraindicated or unacceptable. In line with this perspective, the study population consisted primarily of women in their reproductive years, with a mean age of 35.3 years (± 8.3), representing a clinically relevant demographic in which a fertility-sparing option is a significant concern.

From a reproductive standpoint, progesterone plays a key physiological role in implantation and the maintenance of pregnancy [33]. The favourable safety profile of micronized natural progesterone, at the same dosage (200 mg/day 1 intravaginal capsule/day at bedtime), is well established through its extensive use in obstetric practice [34, 35] for the prevention and management of several pregnancy-related conditions [36], supporting the appropriateness of this approach for symptom control without compromising reproductive potential.

Although pregnancy was not considered a study endpoint and was in fact an exclusion criterion, the occurrence of four pregnancies during intravaginal progesterone treatment is noteworthy. While these observations are purely exploratory and should be interpreted with caution, they further suggest the potential compatibility of this therapeutic approach with reproductive goals and warrant consideration in future studies.

Nevertheless, the results should be interpreted with caution due to the exploratory nature of the study and its inherent limitations. All 65 patients received the same treatment protocol. While all patients completed VAS scores for dysmenorrhea and menorrhagia, only 40 patients provided complete responses regarding dyspareunia and CPP. This limitation should be considered when interpreting the results related to those symptoms.

Despite the promising outcomes, several limitations warrant consideration. The sample size, while adequate for a cohort prospective monocentric study, limits the ability to generalise the findings. The reliance on patient-reported outcomes may introduce reporting bias, potentially overestimating treatment effectiveness. Moreover, loss to follow-up (20/85) and incomplete questionnaires for chronic pelvic pain and dyspareunia (available in 40/65 patients) may introduce attrition bias, which could favour the intervention if patients with poorer outcomes were less likely to complete follow-up. Significantly, the absence of a control group, either placebo or standard therapy, limits the ability to determine the specific therapeutic contribution of intravaginal progesterone. As a result, it is difficult to distinguish the treatment's actual efficacy from a potential placebo effect. This represents a significant limitation of the present study and should be carefully considered when interpreting the clinical outcomes.

## Conclusions

This study provides preliminary evidence that intravaginal micronized natural progesterone (200 mg/day) may represent a feasible non-contraceptive, fertility-sparing option for symptomatic adenomyosis. Treatment seems to be associated with statistically significant improvements across all primary outcomes (dysmenorrhea, AUB, CCP, dyspareunia) with good tolerability and minimal systemic effects. The significant reduction in dyspareunia, alongside other pain symptoms, suggests that targeted local treatment can be effective even for symptoms with complex, multifactorial etiologies. Although pregnancy was not a primary endpoint, the pregnancies observed during the study period suggest that this treatment is compatible with, and may support, reproductive goals in women of childbearing age. Larger controlled studies are warranted to confirm these findings and further define the role of intravaginal progesterone in clinical practice.

## Data Availability

No datasets were generated or analysed during the current study.
